# Adjuvant chemoradiotherapy plus pembrolizumab for locally advanced esophageal squamous cell carcinoma with high risk of recurrence following neoadjuvant chemoradiotherapy: a single-arm phase II study

**DOI:** 10.1007/s00262-024-03826-y

**Published:** 2024-09-09

**Authors:** Jhe-Cyuan Guo, Ta-Chen Huang, Hung-Yang Kuo, Chia-Chi Lin, Feng-Ming Hsu, Jason Chia-Hsien Cheng, Yen-Lin Huang, Min-Shu Hsieh, Pei-Ming Huang, Jang-Ming Lee, Shu-Ling Wu, Chih-Hung Hsu

**Affiliations:** 1 Department of Medical Oncology, National Taiwan University Cancer Center, Taipei, Taiwan; 2 Department of Oncology, National Taiwan University Hospital, Taipei, Taiwan; 3 Graduate Institute of Clinical Medicine, National Taiwan University College of Medicine, Taipei, Taiwan; 4 Graduate Institute of Oncology, National Taiwan University College of Medicine, Taipei, Taiwan; 5 Department of Pathology, National Taiwan University Cancer Center, Taipei, Taiwan; 6 Department of Pathology, National Taiwan University Hospital, Taipei, Taiwan; 7 Department of Surgery, National Taiwan University Hospital, Taipei, Taiwan

**Keywords:** Esophageal squamous cell carcinoma, Adjuvant therapy, Pembrolizumab, High risk, Immune checkpoint inhibitor

## Abstract

**Background:**

Adjuvant nivolumab reduces recurrence in patients with locoregional esophageal cancer who had pathological residual disease after neoadjuvant chemoradiotherapy and R0 resection. However, the efficacy of adjuvant anti-PD-1 therapy in patients at higher risk of recurrence remains unclear.

**Methods:**

This phase II trial (ClinicalTrials.gov identifier: NCT03322267) enrolled patients with locally advanced esophageal squamous cell carcinoma (ESCC) received neoadjuvant chemoradiotherapy plus esophagectomy but still had various risk factors for recurrence, such as involved or close margins (≤ 1 mm), extranodal extension of the involved lymph nodes, and the ypN2-3 stage. Patients received adjuvant therapy composed of a course of cisplatin-based chemoradiotherapy and pembrolizumab (200 mg, IV every 3 weeks) for 18 cycles. The primary endpoint was 1-year relapse-free survival (RFS) rate.

**Results:**

Twenty-five patients were enrolled. The risk factors were tumor margins of ≤ 1 mm (18 patients), extranodal extension of the involved lymph nodes (9 patients), and the ypN2-3 stage (9 patients). The median follow-up duration was 21.6 months (95% CI: 18.7–33.2). The rate of 1-year RFS was 60.0%. The median duration of RFS and overall survival was 14.3 (95% CI: 9.0–19.5) and 21.6 (95% CI: 0.0–45.5) months, respectively. Treatment-emergent adverse events of any grade and those of ≥ 3 grade occurred in 56% and 8% of all patients receiving cisplatin-based chemoradiotherapy and in 79.2% and 12.5% of those receiving pembrolizumab.

**Conclusions:**

Adjuvant chemoradiotherapy followed by pembrolizumab is feasible and may be associated with improved 1-year RFS rate in patients at high risk of recurrence after trimodality therapy for locally advanced ESCC.

*Trial registration number* ClinicalTrials.gov (No. NCT03322267).

**Supplementary Information:**

The online version contains supplementary material available at 10.1007/s00262-024-03826-y.

## Introduction

Esophageal cancer (EC) is a leading malignancy worldwide. According to the GLOBOCAN 2020 database, 604,100 new cases of EC and 544,100 EC-associated deaths have been estimated annually, making EC the eighth most common type of diagnosed malignancy and the sixth most common cause of cancer-related mortality worldwide. EC has 2 major histologic subtypes: esophageal squamous cell carcinoma (ESCC) and esophageal adenocarcinoma (EAC). ESCC accounts for 85% of the total global burden of EC [1].

Although neoadjuvant chemoradiotherapy (CRT) followed by esophagectomy is a standard therapeutic option for patients with locally advanced EC, particularly ESCC, [2, 3] the prognosis of patients with residual pathologic disease after neoadjuvant CRT is unfavorable [4]. A cohort study reported that adjuvant CRT was associated with reduced cancer recurrence and improved survival in patients with locally advanced ESCC having residual disease with advanced pathologic features (ypT3-4 or ypN-positive) after neoadjuvant CRT and esophagectomy [5]. Recently, the CheckMate 577 study—a randomized phase III trial—demonstrated that adjuvant nivolumab, an anti-programmed cell death protein (PD-1) monoclonal antibody, significantly improved disease-free survival (DFS) in patients with locoregional EC or gastroesophageal junction (GEJ) cancer who had pathologic residual disease despite receiving neoadjuvant CRT and R0 resection [6]. The efficacy of adjuvant nivolumab in improving DFS has been reported in both patients with ESCC and those with EAC.

Several pathologic features of post-CRT esophagectomy tissues, such as involved or close resection margins (defined as the presence of vital tumor cells within 1 mm of the proximal, distal, or circumferential resection margin) [7], extranodal extension (ENE) of the involved lymph nodes (LNs) [8], or the ypN2-3 stage [4], are associated with poor prognosis (increased recurrence or reduced survival) in patients with locally advanced ESCC who have received neoadjuvant CRT and undergone esophagectomy. However, the CheckMate 577 study did not include patients with involved or close resection margins. Hence, whether adjuvant anti-PD-1 therapy is beneficial for patients at high risk of cancer recurrence after neoadjuvant CRT, such as those with involved or close resection margins, remains unclear.

Pembrolizumab, a monoclonal antibody against PD-1, is effective in patients with advanced EC, particularly those with ESCC. In the KEYNOTE-181 study, as a second-line systemic therapy, pembrolizumab outperformed chemotherapy in improving clinical outcomes in patients with high expression levels of programmed death-ligand-1 (PD-L1), as indicated by the combined positive scores (CPS) ≥ 10 [9]. Furthermore, the combination of pembrolizumab and chemotherapy outperformed chemotherapy alone in improving clinical outcomes in patients with advanced EC [10]. Multiple clinical trials are underway to evaluate the efficacy of supplementary pembrolizumab treatment for locoregional EC.

We hypothesized that adjuvant therapy with a course of cisplatin-based CRT followed by anti-PD-1 therapy would improve clinical outcomes in patients with locally advanced ESCC who have a high risk of recurrence after neoadjuvant CRT and esophagectomy, and thus conducted this single-arm, phase II trial to investigate the safety and efficacy of adjuvant CRT followed by pembrolizumab treatment. The combination of CRT and subsequent anti-PD-1/anti-PD-L1 treatment has been investigated in multiple cancer types, and has demonstrated significantly increased progression-free survival (PFS) and overall survival (OS) in patients with stage III non–small-cell lung cancer (NSCLC) in PACIFIC trial [11, 12].

## Patients and methods

### Patients

This study included patients (age ≥ 20 years) with histologically confirmed locally advanced ESCC (cT3-4aN0 or cT1-4aN1-3M0 as per the eighth edition of the American Joint Committee on Cancer staging system) who had received neoadjuvant CRT with 40–45 Gy in 20–25 fractions and had subsequently undergone esophagectomy but presented with histologic evidence of pathologic residual disease in a surgical specimen (the esophagus, LNs, or both) and at least 1 risk factor for cancer recurrence (involved or close resection margins [≤ 1 mm], ENE of the involved LNs, or ypN2-3 stage). In addition, we included patients with an Eastern Cooperative Oncology Group performance status of 0 or 1, adequate organ function, and adequate contraception during study period. The key exclusion criteria were as follows: adenocarcinoma of the esophagus or GEJ; synchronously diagnosed with a squamous cell carcinoma (other than EC) of the upper aerodigestive way, presence of active autoimmune disease or any other condition necessitating the chronic use of systemic corticosteroids or immunosuppressive agents; or a history of primary immunodeficiency, active pulmonary tuberculosis, noninfectious pneumonitis, or organ transplantation. In addition, we excluded patients had not recovered from an adverse event resulting from previous treatment; those with active infection necessitating systemic therapy; those with known psychiatric or substance abuse disorders; those who had previously received anti-PD-1, anti-PD-L1, or anti-PD-L2 agents; and those who had received a live vaccine within 30 days of the planned initiation date of the protocol therapy.

### Study design and treatment

This single-arm, single-center, open-label phase II investigator-initiated trial (ClinicalTrials.gov identifier: NCT03322267) was designed by the lead investigators at National Taiwan University Hospital and funded by Merck Sharp & Dohme. The present study was conducted in accordance with the ethical principles of the Declaration of Helsinki and the Good Clinical Practice guidelines. The study protocol was approved by the research ethics committee of our institute (approval number: 201708042MIPD). Written informed consent was obtained from all patients before screening and enrollment. Clinical data were collected by the investigators and research staff. The authors had full access to the study data. All authors have contributed to this study and reviewed the manuscript before its submission for publication.

The adjuvant therapy administered in this trial involved a course of cisplatin-based CRT and pembrolizumab treatment. In the adjuvant CRT phase, cisplatin (30 mg/m^2^) was intravenously administered to the patients every week for 2 cycles; simultaneously, radiotherapy (18–26 Gy) was administered in 10 to 13 fractions (180–200 cGy per fraction) 5 days a week to a cumulative dose of 60–66 Gy delivered by intensity-modulated or volumetric-modulated radiotherapy technique. The target volume encompassed the primary esophageal tumor bed and the metastatic nodal basin(s) with adequate margins to account for the risk subclinical disease plus a 5 mm margin for the planning target volume. The maximal cumulative dose for the spinal cord was 50 Gy in equivalent dose of 2 Gy per fraction (EQD2) and no more than one-third of heart receiving more than 60 Gy of the cumulative dose in EQD2. This combination therapy was initiated within 8 to 12 weeks after esophagectomy. Radiotherapy was delivered using megavoltage linear accelerators (≥ 6 megavoltage photon) by using the multiple field (≥ 2) technique. Simulation computed tomography was performed to evaluate the isodose distribution, and dose-volume histograms were generated. The clinical target volume was set considering the regions at risk (eg, the primary tumor bed with involved or close resection margins) and metastatic nodal stations with the ENE of the involved LNs or the ypN2-3 stage.

Adjuvant pembrolizumab (200 mg flat dose) was intravenously administered every 3 weeks for 18 cycles, but it was discontinued upon the occurrence of unacceptable adverse events, relapse of cancer, or withdrawal of consent. This treatment was started after the patients had recovered from adjuvant cisplatin-based CRT.

### Patient assessment

All patients underwent physical examinations and tumor assessments at baseline. Computed tomography (with/without contrast) was performed for the neck, chest, and abdomen 28 days before the initiation of protocol therapy. To monitor potential tumor recurrence, all patients were subjected to pertinent evaluation and imaging studies; the assessments were conducted after every 4 cycles of pembrolizumab treatment in the treatment phase, followed by every 3 months in the first and second years after treatment and every 6 months in third and fourth years after treatment. Patients who discontinued the treatment for reasons other than disease recurrence were evaluated at posttreatment follow-ups to monitor their disease status until the recurrence of disease, initiation of new anti-cancer treatment, withdrawal of consent for participation, or failure to attend further follow-up appointments. All patients were followed up (through phone calls or in-person visits) for OS until the occurrence of death, withdrawal of consent, or end of the study, whichever occurred first. After the end of the protocol treatment, each patient was followed up for 30 days to investigate the development of potential adverse events. Serious adverse events were monitored for 90 days after the end of the protocol treatment; however, in patients who started receiving a new anticancer therapy, adverse events were monitored for 30 days after the end of the protocol treatment.

### Endpoints

The primary endpoint was 1-year relapse-free survival (RFS). The secondary endpoints were the median duration of RFS and OS, rates of 3-year RFS and OS, and toxicity and safety of the protocol treatment. Exploratory end points were the correlations of RFS and OS with PD-L1 expression. Disease relapse was defined as the development of suspicious lesions, as detected in imaging studies (according to the Response Evaluation Criteria In Solid Tumours (RECIST) version 1.1). Histologic confirmatory diagnoses were conducted, if feasible, for patients with suspicious lesions. Adverse events were monitored and graded throughout the treatment and follow-up periods according to the National Cancer Institute Common Terminology Criteria for Adverse Events (version 4.0).

### PD-L1 expression

Formalin-fixed, paraffin-embedded surgical tissue specimens were cut into 4-μm-thick sections and stained with Dako PD-L1 IHC 22C3 pharmDx (Dako, Carpinteria, CA, USA). The expression levels of PD-L1 in tumor tissues was evaluated through immunohistochemical staining, which was performed using the PD-L1 Clone 22C3 pharmDx Kit and the Automated Link 48 platform (Dako). PD-L1 was assessed on the basis of the CPS, which was calculated as follows: (number of PD-L1-positive cells [tumor cells, macrophages, and lymphocytes]/total number of tumor cells) × 100 [13].

### Tumor-infiltrating lymphocytes

Formalin-fixed, paraffin-embedded surgical tissue specimens were cut into 4-μm-thick sections and stained with hematoxylin and eosin. Tumor-infiltrating lymphocytes (TILs) in solid tumors were scored separately for TILs in the intratumoral compartment and TILs in the stromal compartment [14].

### Sample size calculation

On the basis of the results of a subgroup analysis performed in our previous study [4], we determined the median RFS for locally advanced ESCC patients who harbored risk factors for recurrence, including involved or close resection margins [≤ 1 mm], ENE of the involved LNs, or ypN2-3 stage, after neoadjuvant CRT followed by esophagectomy to be 6.7 months; this was equivalent to a 1-year RFS rate of 32.3%. To attain the improvement of 1-year RFS rate to 60% (from 32%), the sample size of 24 was required through an exact binomial test with 80% power and 5% significance. The estimated time for patient accrual was 2 years. In addition, a minimum duration of 3 years was required to complete the follow-up of the last accrued patient. The projected duration of the study was 5 years.

### Statistical analysis

Data locking was performed on May 27, 2024. Statistical analyses were performed using the intention-to-treat principle. Descriptive statistics was calculated to analyze baseline characteristics and adverse events. RFS was defined as the interval between patient enrollment to disease recurrence, death, or the last follow-up (censored). OS was defined as the interval between patient enrollment and death due to any cause or the last follow-up (censored). Kaplan–Meier curves were generated for survival analyses, and a log-rank test as well as restricted mean survival time (RMST) with 3-year timepoint [15] were used to compare survival between patients stratified by the expression levels of PD-L1 and between those stratified by different prognostic factors. All statistical analyses were performed using Prism (version 5.01; GraphPad, San Diego, CA, USA) and MedCalc.

## Results

### Patient characteristics

**Fig. 1:**
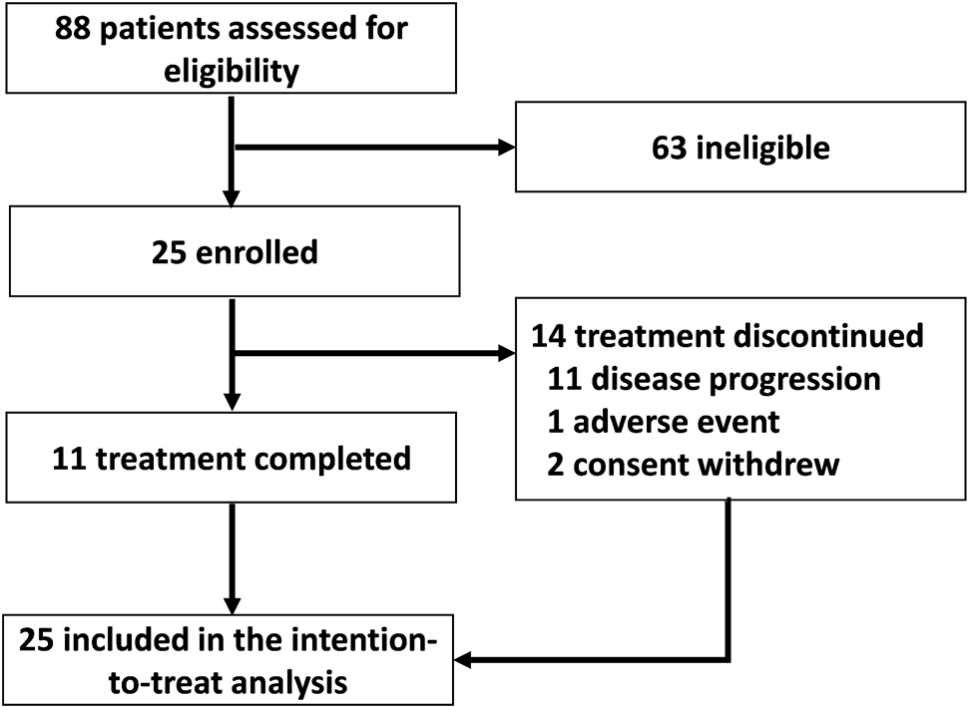
Study cohort and patient deposition

Among the 88 patients screened at National Taiwan University Hospital, Taipei City, Taiwan, between November 2018 and December 2022, 25 were included in the present study. The data of the patients who had received any of the protocol therapies at our hospital were included in the intention-to-treat analysis (Fig. 1). Most of the included patients were men (88%), were current smokers (84%), and had primary tumors located in the mid-thoracic esophagus (72%). Despite the administration of neoadjuvant CRT (median radiation dose: 45 Gy), the pathologic stage in most patients was ypT3 (52%), ypN-positive (76%), and ypStage III (68%). A total of 18 patients had only 1 pathological risk factor for recurrence (72%), whereas 7 patients had ≥ 2 risk factors (28%). The patients’ baseline characteristics are summarized in Table 1.

**Table 1:** Baseline Characteristics (N = 25)

Characteristics	No. (%)
Median age, years (range)	62.3 (43.3–73.1)
ECOG performance status	
0	8 (32)
1	17 (68)
Gender	
Male	22 (88)
Female	3 (12)
Smoking	
Current	21 (84)
Never	4 (16)
Primary esophageal cancer	
Cervical	1 (4)
Upper thoracic	3 (12)
Middle thoracic	18 (72)
Lower thoracic	3 (12)
Median interval between operation and enrollment, days (range)	34 (23–102)
Clinical stage before nCRT	
II	1 (4)
III	20 (80)
IVA	4 (16)
Chemotherapy regimen of nCRT	
Cisplatin and 5-FU	14 (56)
Cisplatin or carboplatin and paclitaxel	11 (44)
Median radiation dose of nCRT, Gy (range)	45 (40–50)
Pathologic stage after nCRT	
IB	1 (4)
II	7 (28)
IIIA	5 (20)
IIIB	12 (48)
Median number of dissected lymph node (range)	44 (18–63)
Tumor regression grade (TRG)	
TRG 2 (1 ~ 10% residual carcinoma)	7 (28)
TRG 3 (11 ~ 50% residual carcinoma)	13 (52)
TRG 4 (> 50% residual carcinoma)	5 (20)
Risk factor	
Margin ≤ 1 mm	18 (72)
Extracapsular invasion	9 (36)
ypN2-3	9 (36)
Post-nCRT PD-L1 expression	
CPS < 10	15 (60)
CPS ≥ 10	10 (40)

*CPS* combined positive score, *ECOG* Eastern cooperative oncology group, *nCRT* neoadjuvant chemoradiotherapy, *PD-L1* programmed cell death ligand 1

### Survival rates

**Fig. 2:**
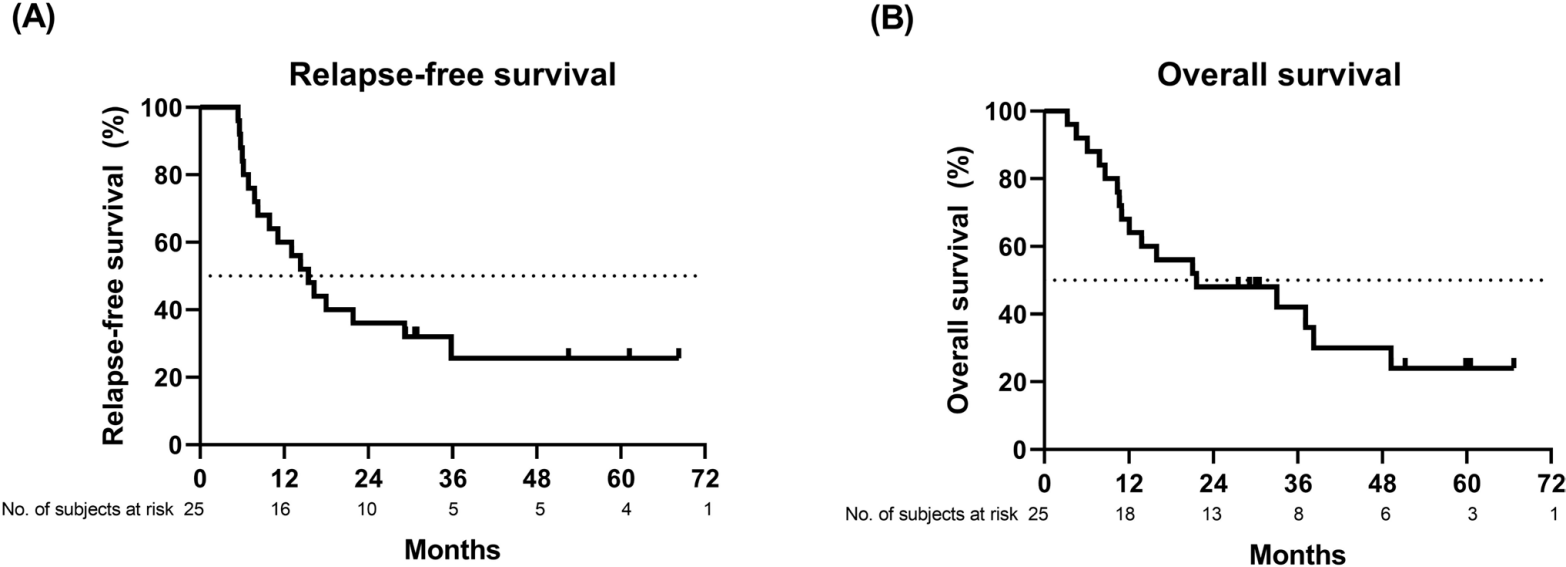
Kaplan–Meier survival curves of relapse-free survival (**A**) and overall survival (**B**) of the entire cohort

The median follow-up duration was 21.6 months (95% confidence interval [CI]: 18.7–33.2). The rate of 1-year RFS was 60.0% (Fig. 2A). The median durations of RFS and OS were 14.3 (95% CI: 9.0–19.5) and 21.6 (95% CI: 0.0–45.5) months, respectively (Fig. 2). The rates of 3-year RFS and OS were 25.2% and 42.0%, respectively.

**Fig. 3:**
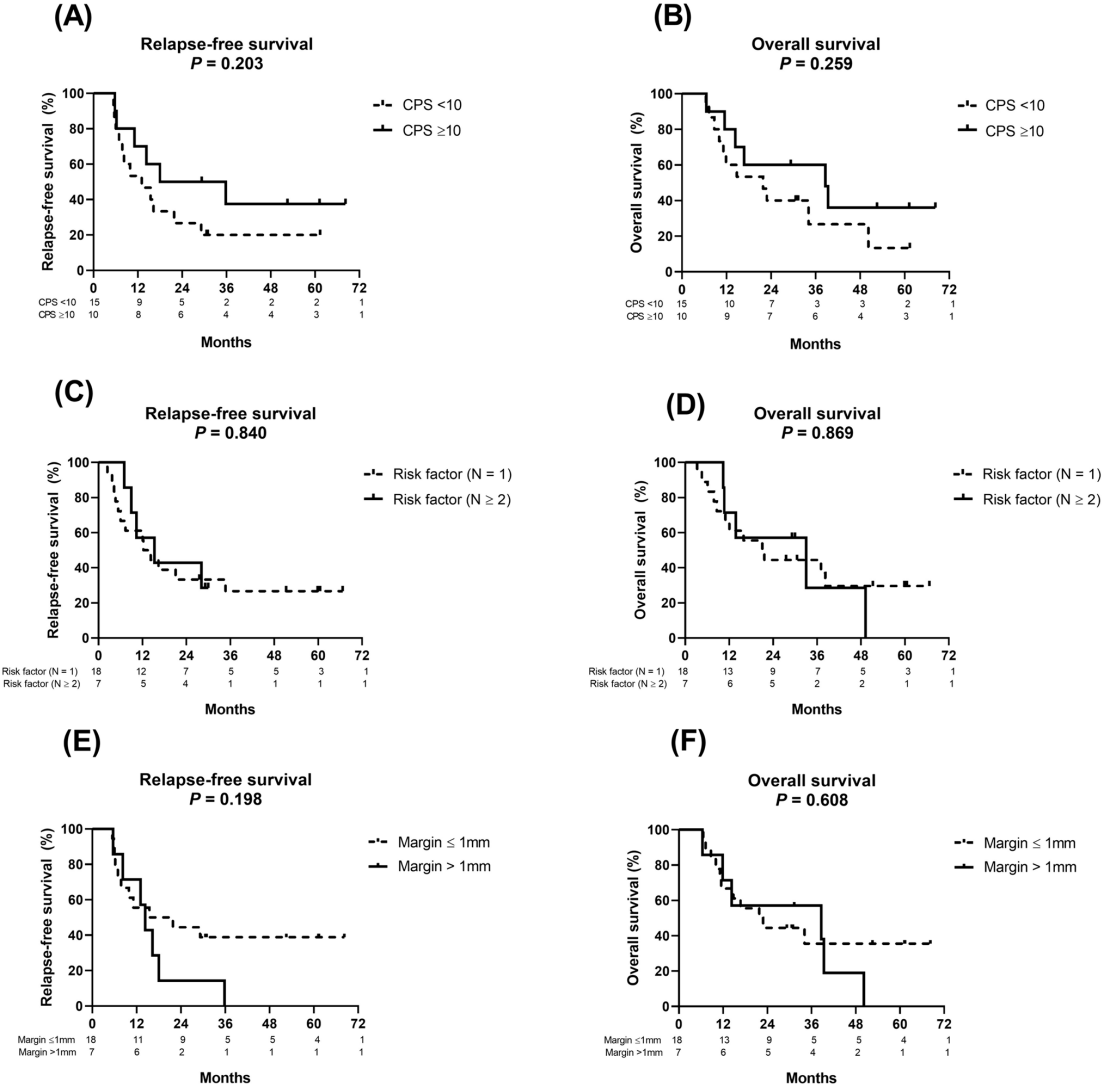
Kaplan–Meier survival curves of relapse-free survival and overall survival of patients classified by PD-L1 expression (combined positive score, CPS ≥ 10 versus < 10) (**A**, **B**), risk factor number (**C**, **D**) and margin status (**E**, **F**)

We observed a numerically longer median RFS and OS among patients with high expression levels of PD-L1 (i.e., CPS ≥ 10) in tumor tissues than among those with low expression levels of PD-L1 (RFS: 26.9 vs 13.0 months, hazard ratio [HR]: 0.54 [95% CI: 0.214–1.361] [*P* = 0.203]; OS: 38.7 vs 21.8 months, HR: 0.57 [95% CI: 0.222–1.485] [*P* = 0.259]; Fig. 3A, B).

No significant difference was observed in the median duration of RFS or OS between the patients with 1 risk factor and those with ≥ 2 risk factors (RFS: 13.3 vs 15.3 months, HR: 0.90 [95% CI: 0.329–2.470] [*P* = 0.840]; OS: 21.3 vs 33.0 months, HR: 1.09 [95% CI: 0.377–3.160] [*P* = 0.869]; Fig. 3C, D) or between the patients with involved or close margins and those without involved or close margins (RFS: 18.6 vs 14.3 months, HR: 1.83 [95% CI: 0.637–5.269] [*P* = 0.198]; OS: 22.3 vs 38.7 months, HR: 1.30 [95% CI: 0.457–3.666] [*P* = 0.608]; Fig. 3E, F).

**Fig. 4:**
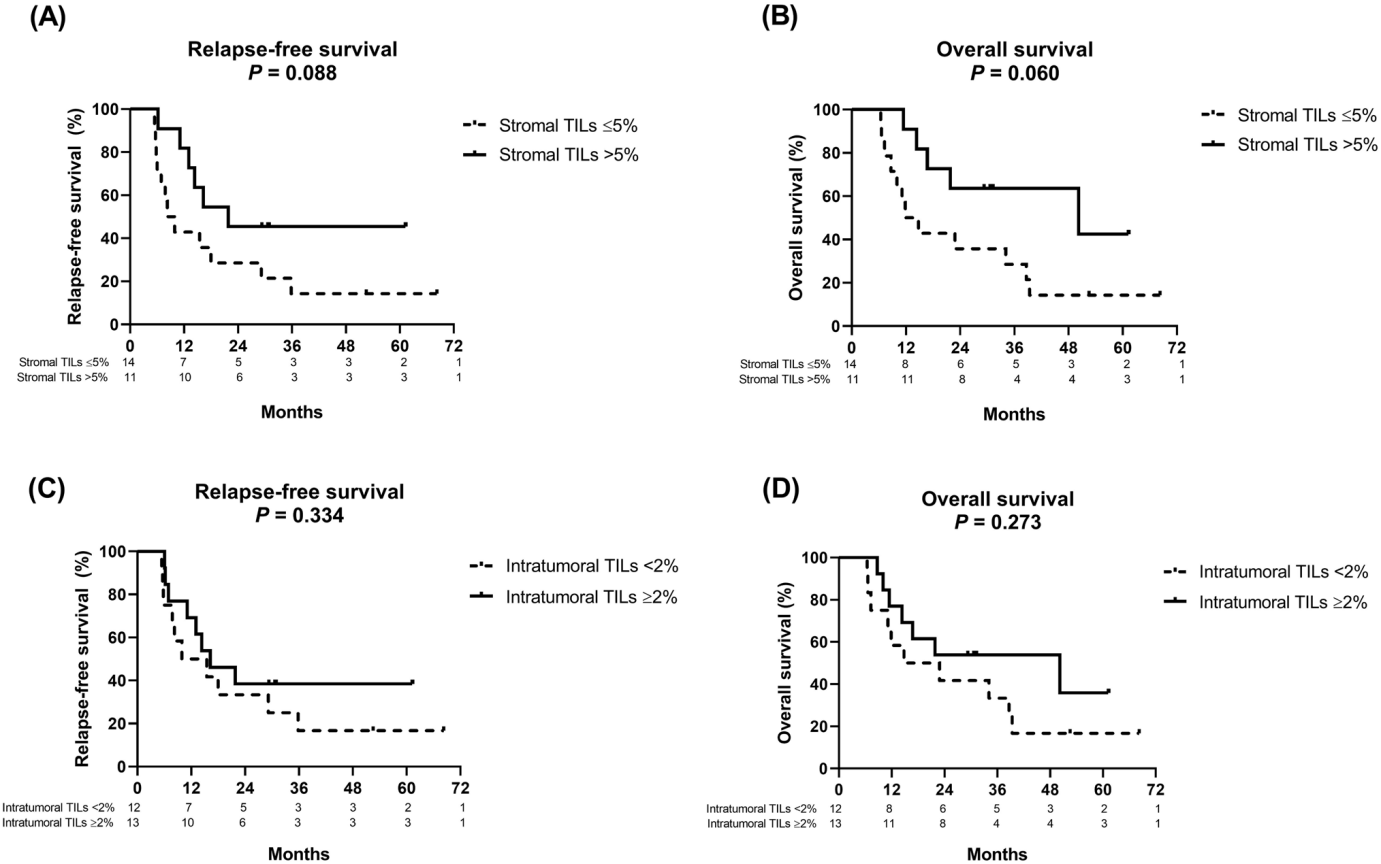
Kaplan–Meier survival curves of relapse-free survival and overall survival of patients classified by stromal tumor-infiltrating lymphocytes (TILs) (> 5% versus ≤ 5%) (**A**, **B**) and intratumoral TILs (≥ 2% versus < 2%) (**C**, **D**)

A trend toward improvement in the median duration of RFS and the median duration of OS were noted among patients with a high abundance of stromal TILs and those with a low abundance of stromal TILs (RFS: 21.8 vs 9.1 months, HR: 0.44 [95% CI: 0.174–1.107] [*P* = 0.088]; OS: 50.2 vs 13.3 months, HR: 0.38 [95% CI: 0.148–0.994] [*P* = 0.060]; Fig. 4A, B). However, no significant difference was observed in the median duration of RFS or OS between patients with a high abundance of intratumoral TILs and those with a low abundance of intratumoral TILs (RFS: 16.3 vs 12.7 months, HR: 0.64 [95% CI: 0.251–1.612] [*P* = 0.334]; OS: 50.2 vs 18.8 months, HR: 0.59 [95% CI: 0.227–1.527] [*P* = 0.273]; Fig. 4C, D). We also used another method, the RMST with 3-year timepoint analysis, to evaluate the survival differences between different subgroups. The analysis demonstrated very similar results (Fig. S1), i.e., the RMSTs of RFS and OS were numerically longer in patients with CPS ≥ 10 than those with CPS < 10, the RMST of RFS was numerically longer in patients with a high abundance of stromal TILs than those with a low abundance of stromal TILs, and the RMST of OS was significantly longer in patients with a high abundance of stromal TILs than those with a low abundance of stromal TILs.

### Treatment exposure and toxicity

All patients, except 1 patient, received per-protocol therapy of cisplatin-based CRT (cisplatin: 30 mg/m^2^, intravenously administered every week for 2 cycles; radiotherapy: 18–26 Gy, delivered in 10–13 fractions [180–200 cGy per fraction]). In the exception case, the patient received 4 cycles of cisplatin-based CRT (dose: approximately 47 Gy; delivered in 16 fractions) targeting the tumor bed and mediastinal LNs, given the small (short axis < 1 cm) mediastinal LNs noted on baseline computed tomography. One patient withdrew consent after cisplatin-based CRT and hence did not receive any dose of pembrolizumab, and another patient withdrew consent after 4 cycles of pembrolizumab treatment. The median number of pembrolizumab treatment cycles was 9 (interquartile range: 4–18).

Table 2 presents the incidence rates of adverse events. Treatment-emergent adverse events (TEAEs; any grade) occurred in 56% of all patients receiving cisplatin-based CRT and in 79.2% of those receiving pembrolizumab treatment. Most of these TEAEs were of low grade; nonetheless, grade ≥ 3 TEAEs occurred in 8% of all patients receiving cisplatin-based CRT and 12.5% of those receiving pembrolizumab treatment. The most common TEAEs in patients receiving cisplatin-based CRT were bone marrow toxicities, including leucopenia (36%), anemia (20%), and thrombocytopenia (12%). Grade ≥ 3 TEAEs in patients receiving cisplatin-based CRT included leucopenia (4%) and pancreatitis (4%). The most common TEAEs in patients receiving pembrolizumab treatment were infection (33.3%), diarrhea (29.2%), pneumonitis (25%), and fatigue (20.8%). Pneumonitis was differentiated with infection by excluding possible respiratory pathogen and was confirmed by spontaneously resolved or improved after systemic steroid use. Grade ≥ 3 TEAEs in patients receiving pembrolizumab treatment were colitis (4.2%) and infection (8.3%).

**Table 2:** Treatment emergent adverse events

Adverse event	Cisplatin-CRT (N = 25)		
	Any Grade N (%)	Grade 3 N (%)	Grade 4 N (%)
Any adverse event	14 (56)	2 (8)	0 (0)
Leucopenia	9 (36)	1 (4)	0 (0)
Anemia	5 (20)	0 (0)	0 (0)
Thrombocytopenia	3 (12)	0 (0)	0 (0)
Anorexia	2 (8)	0 (0)	0 (0)
Nausea	2 (8)	0 (0)	0 (0)
Vomiting	2 (8)	0 (0)	0 (0)
Constipation	2 (8)	0 (0)	0 (0)
Neutropenia	1 (4)	0 (0)	0 (0)
Lymphopenia	1 (4)	0 (0)	0 (0)
Hypokalemia	1 (4)	0 (0)	0 (0)
Diarrhea	1 (4)	0 (0)	0 (0)
Pancreatitis	0 (0)	1 (4)	0 (0)
Neurotoxicity	0 (0)	0 (0)	0 (0)
Nephrotoxicity	0 (0)	0 (0)	0 (0)
Ototoxicity	0 (0)	0 (0)	0 (0)

Adverse event	Pembrolizumab (N = 24)		
	Any grade N (%)	Grade 3 N (%)	Grade 4 N (%)
Any adverse event	19 (79.2)	3 (12.5)	0 (0)
Infection^α^	8 (33.3)	2 (8.3)	0 (0)
Diarrhea	7 (29.2)	0 (0)	0 (0)
Pneumonitis	6 (25.0)	0 (0)	0 (0)
Fatigue	5 (20.8)	0 (0)	0 (0)
Elevated AST/ALT	4 (16.7)	0 (0)	0 (0)
Hypothyroidism	3 (12.5)	0 (0)	0 (0)
Skin rash	3 (12.5)	0 (0)	0 (0)
Pruritus	2 (8.3)	0 (0)	0 (0)
Pyrexia	2 (8.3)	0 (0)	0 (0)
Nausea	2 (8.3)	0 (0)	0 (0)
Colitis	2 (8.3)	1 (4.2)	0 (0)
Anorexia	1 (4.2)	0 (0)	0 (0)
Arthritis	1 (4.2)	0 (0)	0 (0)
Anemia	1 (4.2)	0 (0)	0 (0)
Headache	1 (4.2)	0 (0)	0 (0)
Hypomagnesemia	1 (4.2)	0 (0)	0 (0)

Infection: herpes zoster, pneumonia, Clostridium difficile related colitis, SARS-CoV-2 infection, and Pneumocystis jirovecii pneumonia

## Discussion

Before the CheckMate 577 study, various adjuvant therapies using chemotherapy and radiotherapy were commonly administered to patients with locally advanced EC who had pathologic residual disease after neoadjuvant CRT plus surgery; this strategy was prevalent despite lack of high-level clinical evidence [16]. The CheckMate 577 study demonstrated that 1-year adjuvant therapy with nivolumab significantly improved DFS in patients with stage II/III EC or GEJ cancer who had pathologic residual disease after receiving neoadjuvant CRT and R0 resection [6]. However, the study did not include patients with involved or close resection margins, which are a well-known risk factor for recurrence. Whether adjuvant anti-PD-1 therapy can benefit this group of patients at high risk of recurrence remains unclear. Thus, we conducted this single-arm phase II trial in patients with locally advanced ESCC, who have a higher risk of recurrence than did the patients in the CheckMate 577 study. We demonstrated that a short-term cisplatin-based CRT followed by pembrolizumab treatment increased the rate of 1-year RFS compared with the rate in a historical control group.

One previous retrospective study demonstrated a survival benefit associated with adjuvant CRT compared with observation for patients with locally advanced ESCC harboring ypT3-4 or ypN + disease after trimodality therapy [5]. The results of the current study are in line with the aforementioned retrospective study and support that additional therapy is needed to improve the outcomes of ESCC patients with high risk for recurrence despite treated with trimodality therapy for their locally advanced disease. However, the exact contribution of pembrolizumab and CRT to the improved RFS seen in our patient cohort cannot be assured because our study is a single-arm non-comparable clinical trial.

Our study enrolled patients who had unfavorable pathological features with high risk for recurrence, especially involved or close resection margins (≤ 1 mm) in their post-CRT, postoperative tumor tissues (in 72% of enrolled patients). Compared with CheckMate 577 and other reported studies investigating the efficacy of adjuvant anti-PD-1 or anti-PD-L1 therapy for patients with locally advanced EC who received neoadjuvant CRT [6, 17, 18], patients enrolled in our study had other unfavorable pathologic features, such as the ypT3 (52%), ypN-positive (76%; ypN1, 40%; ypN2, 36%), and ypStage III (68%) stages [6, 17, 18]. Despite having multiple risk factors for poor prognosis, our patients exhibited a 1-year RFS rate similar to that reported in the CheckMate 577 study [6] and similar to that in a phase II trial investigating the efficacy of adjuvant durvalumab, a monoclonal antibody against PD-L1, in patients with locally advanced EAC or GEJ adenocarcinoma after neoadjuvant CRT and surgery [17].

In a recent randomized phase II study, the efficacy of adjuvant therapy with durvalumab versus placebo was investigated in patients with ESCC who underwent curative surgery after neoadjuvant CRT [18]. However, the study did not find DFS or OS benefit in patients receiving adjuvant durvalumab. The discrepancy in the outcomes of adjuvant anti-PD-1 and anti-PD-L1 therapy between the aforementioned randomized phase II study and the CheckMate 577 study might be because of several factors. First, the randomized phase II study included patients with pathologic complete response (pCR, 33%) and non-pCR (67%) after neoadjuvant CRT; whereas, the CheckMate 577 study did not include patients with pCR after neoadjuvant CRT. The improved prognosis in patients with pCR might have diluted the potential benefits of adjuvant therapy. Second, the randomized phase II study included a total of 86 patients. The sample size was small, and the results may have been affected by patient selection bias [18].

Pembrolizumab has been incorporated into the therapeutic landscape for patients with advanced EC, particularly those with ESCC. In the KEYNOTE-180, KEYNOTE-181, and KEYNOTE-590 studies, patients with high expression levels of PD-L1 (CPS ≥ 10) benefitted significantly more from pembrolizumab than patients with low expression levels of PD-L1. In the present study, patients with high expression levels of PD-L1 (CPS ≥ 10) tended to have longer RFS than did those with low expression levels of PD-L1 (CPS < 10). This finding is in line with those of other studies assessing the efficacy of adjuvant nivolumab or durvalumab [6, 17, 18]. We further found that a higher abundance of stromal TILs in postoperative tumor tissues was associated with longer RFS and OS. This finding corroborates that of a biomarker study involving patients with advanced ESCC who received nivolumab [19]. Similar to our study, the aforementioned study reported an association between a high abundance of stromal TILs and the long-term benefits of anti-PD-1 therapy [19].

In the present study, patients with ≥ 2 risk factors had RFS and OS similar to those of patients with a single risk factor. This finding might be because of the small number of patients in each group in our study and should be investigated in future studies involving large cohorts of patients with various risk factors for cancer recurrence.

Most of our patients experienced at least 1 TEAE (of any grade) after receiving cisplatin-based CRT (56%) and pembrolizumab treatment (79.2%). However, only 8% and 12.5% of all patients receiving CRT and pembrolizumab treatment, respectively, developed grade ≥ 3 TEAEs. Overall, we noted no new or unexpected TEAEs. The incidence rate of pneumonitis (25% for all grades of pneumonitis) in our study was higher than that noted with pembrolizumab monotherapy in the historical control (< 10% for all grades) group in the KEYNOTE-180 and KEYNOTE-181 studies [9, 13]. The increased incidence of pneumonitis in the patients receiving pembrolizumab treatment in our trial may be attributed to that fact that all of the included patients had been exposed to thoracic irradiation during the administration of neoadjuvant and adjuvant CRT [20]. Similar findings have been reported in clinical trials involving patients with NSCLC: the incidence rate of pneumonitis was higher in patients receiving CRT followed by durvalumab (in the PACIFIC study) than in those receiving durvalumab monotherapy (in the MYSTIC study) [11, 21]. The increased risk of pneumonitis should be considered in future clinical trials evaluating the efficacy of the combination of anti-PD-1 or anti-PD-L1 therapy with radiotherapy in patients with thoracic malignancies, such as EC.

Our study has several limitations. First, the sample size was small; the trial included only 25 patients with ESCC, and did not include a control arm. Nevertheless, despite the small sample size, our findings revealed the feasibility and probable potential benefits of adjuvant cisplatin-based CRT followed by pembrolizumab treatment for patients with locally advanced ESCC, who are at a high risk of recurrence after neoadjuvant CRT; these patients are not well-represented in the literature on adjuvant therapy. Second, whether an improvement in RFS can be translated into an improvement in OS remains unknown. Furthermore, whether RFS or DFS can be regarded as a surrogate endpoint for OS requires further investigation. A trial-level meta-analysis demonstrated DFS as a surrogate endpoint for OS in patients with resectable EC, GEJ cancer, or gastric cancer after neoadjuvant and perioperative therapy [22]. The OS results from the CheckMate 577 study may clarify whether improved RFS can be translated into improved OS in the future. Third, recent success of ESOPEC and JCOG1109 studies, demonstrating that neoadjuvant chemotherapy using 3-drug combination, might change the therapeutic landscape of locally EC including ESCC [23, 24]. However, the role of adjuvant anti-PD-1 therapy in localized EC following neoadjuvant chemotherapy or perioperative chemotherapy remains unclear. Currently, multiple studies are ongoing to address the roles of chemotherapy with/without anti-PD-1 therapy, administered in either neoadjuvant setting or perioperative setting, in patients with locoregional EC or ESCC.

## Conclusions

Adjuvant therapy with a short course of cisplatin-based CRT followed by pembrolizumab treatment for one year may benefit patients with pathologic residual disease who are at a risk of recurrence (involved or close margin [≤ 1 mm], ENE of the involved LNs, or ypN2-3 stage) after receiving neoadjuvant CRT for locally advanced ESCC. The therapeutic strategy adopted in this trial increased the 1-year RFS rate compared with the rate in the historical control cohort. The safety profile of this therapeutic strategy is consistent with those reported in relevant studies. High expression levels of PD-L1 (defined as CPS ≥ 10) and a high abundance of stromal TILs may be associated with the efficacy of pembrolizumab in our patient population. Further studies are warranted to validate the findings of the present study.

## Supplementary Information

Below is the link to the electronic supplementary material.

Supplementary file1 (PDF 789 KB)

## Data Availability

No datasets were generated or analysed during the current study.
