# Supplementary material for: Adjuvant chemoradiotherapy plus pembrolizumab for locally advanced esophageal squamous cell carcinoma with high risk of recurrence following neoadjuvant chemoradiotherapy: a single-arm phase II study
Source: Cancer Immunol Immunother. 2024 Sep 9;73(11):230. doi: 10.1007/s00262-024-03826-y (PMC11383884; doi:10.1007/s00262-024-03826-y)
Supplement: Supplementary file 1 — Supplementary file1 (PDF 789 KB) [file 262_2024_3826_MOESM1_ESM.pdf]

Figure S1. Restricted mean survival time (RMST) with 3-year landmark analysis

Relapse-free survival (RFS)

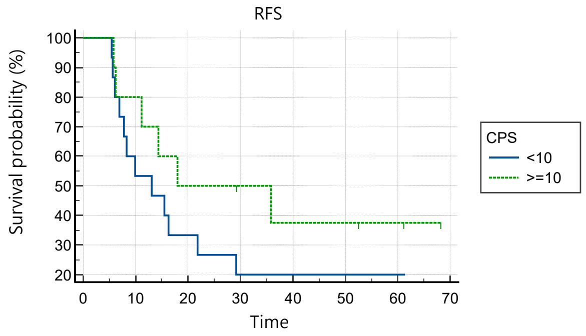

Number at risk

Group								
Group: <10	15	8	5	3	1	1	1	0
Group: >=10	10	8	5	4	3	3	2	0

RMST (CPS <10 vs. ≥10):
16.9 months vs. 23.5 months (P = 0.187)

Overall survival (OS)

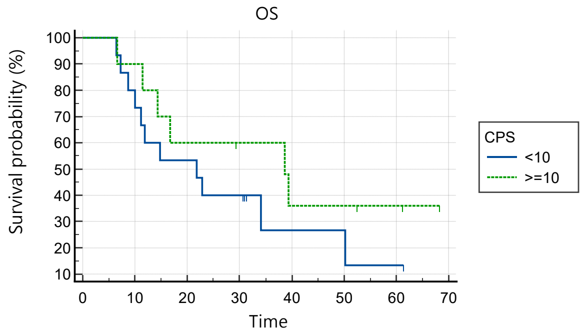

Number at risk

Group								
Group: <10	15	11	8	6	2	2	1	0
Group: >=10	10	9	6	5	3	3	2	0

RMST (CPS <10 vs. ≥10):
21.8 months vs. 26.5 months (P = 0.299)

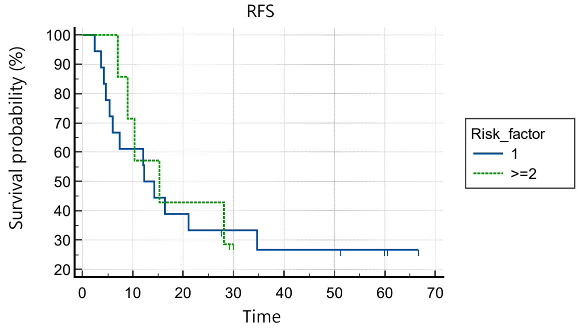

Number at risk

Group								
Group: 1	18	11	7	5	4	4	2	0
Group: >=2	7	5	3	0	0	0	0	0

RMST (risk factor = 1 vs. ≥2):
16.1 months vs. 18.5 months (P = 0.587)

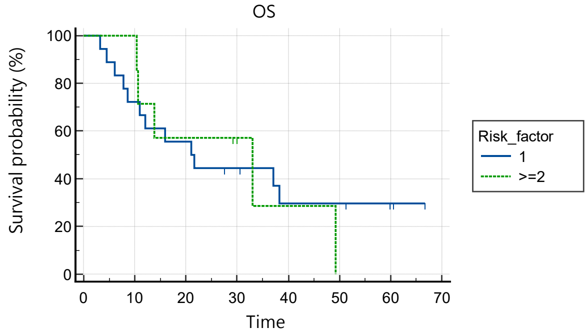

Number at risk

Group								
Group: 1	18	13	10	7	4	4	2	0
Group: >=2	7	7	4	2	1	0	0	0

RMST (risk factor = 1 vs. ≥2):
22.2 months vs. 24.7 months (P = 0.611)

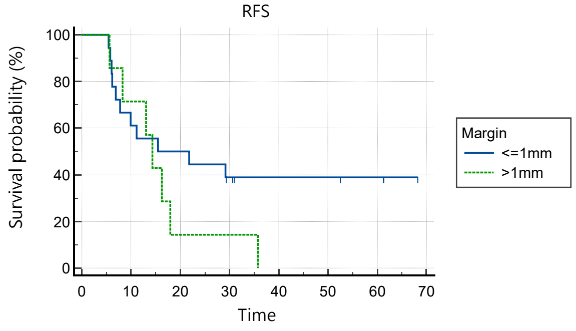

Number at risk

Group								
Group: <=1mm	18	11	9	6	4	4	3	0
Group: >1mm	7	5	1	1	0	0	0	0

RMST (margin >1 mm vs. ≤1mm):
15.9 months vs. 20.9 months (P = 0.280)

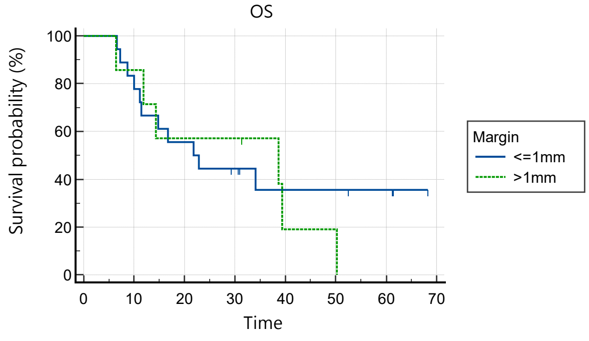

Number at risk

Group								
Group: <=1mm	18	14	10	7	4	4	3	0
Group: >1mm	7	6	4	4	1	1	0	0

RMST (margin >1 mm vs. ≤1mm):
25.2 months vs. 23.1 months (P = 0.682)

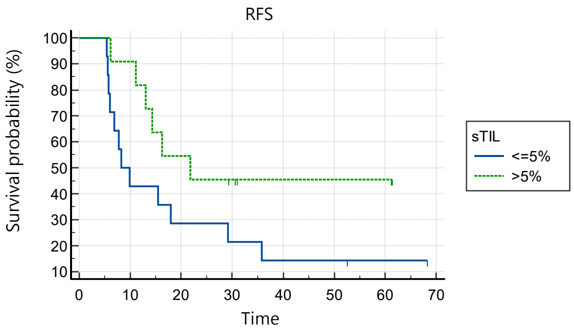

Number at risk
Group: <=5%
14 6 4 3 2 2 1 0
Group: >5%
11 10 6 4 2 2 2 0

RMST (sTIL >5% vs. ≤5%):
23.9 months vs. 16.1 months (P = 0.102)

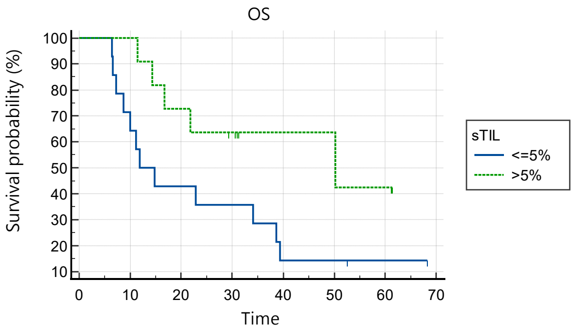

Number at risk
Group: <=5%
14 9 6 5 2 2 1 0
Group: >5%
11 11 8 6 3 3 2 0

RMST (sTIL >5% vs. ≤5%):
28.8 months vs. 19.8 months (P = 0.031)

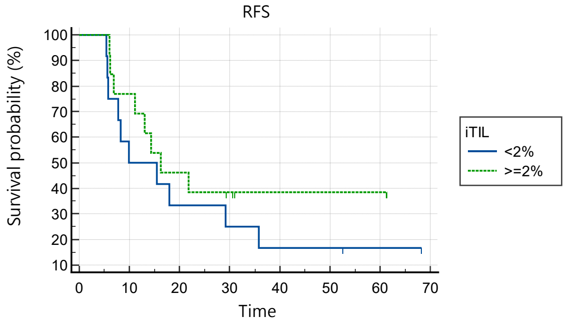

Number at risk
Group: <2%
12 6 4 3 2 2 1 0
Group: >=2%
13 10 6 4 2 2 2 0

RMST (iTIL ≥2% vs. <2%):
21.2 months vs. 17.8 months (P = 0.484)

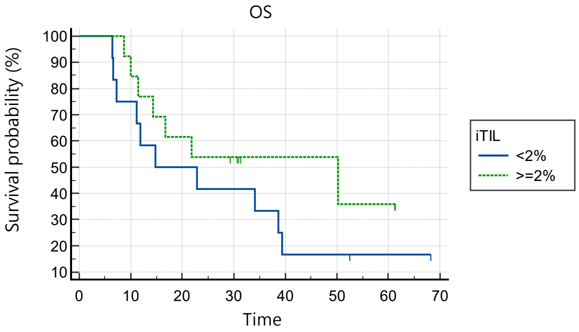

Number at risk
Group: <2%
12 9 6 5 2 2 1 0
Group: >=2%
13 11 8 6 3 3 2 0

RMST (iTIL ≥2% vs. <2%):
25.8 months vs. 21.6 months (P = 0.353)
